# Structure, thermodynamics, and rearrangement mechanisms in gold clusters—insights from the energy landscapes framework†
†Electronic supplementary information (ESI) available. See DOI: 10.1039/c7nr07123j


**DOI:** 10.1039/c7nr07123j

**Published:** 2017-12-15

**Authors:** D. Schebarchov, F. Baletto, D. J. Wales

**Affiliations:** a University Chemical Laboratories , Lensfield Road , Cambridge CB2 1EW , UK . Email: Dmitri.Schebarchov@gmail.com ; Email: dw34@cam.ac.uk; b Department of Physics , King's College London , London WC2R 2LS , UK . Email: francesca.baletto@kcl.ac.uk

## Abstract

We consider finite-size and temperature effects on the structure of model Au_N_ clusters (30 ≤ *N* ≤ 147) bound by the Gupta potential. Equilibrium behaviour is examined in the harmonic superposition approximation, and the size-dependent melting temperature is also bracketed using molecular dynamics simulations. We identify structural transitions between distinctly different morphologies, characterised by various defect features. Reentrant behaviour and trends with respect to cluster size and temperature are discussed in detail. For *N* = 55, 85, and 147 we visualise the topography of the underlying potential energy landscape using disconnectivity graphs, colour-coded by the cluster morphology; and we use discrete path sampling to characterise the rearrangement mechanisms between competing structures separated by high energy barriers (up to 1 eV). The fastest transition pathways generally involve metastable states with multiple fivefold disclinations and/or a high degree of amorphisation, indicative of melting. For *N* = 55 we find that reoptimising low-lying minima using density functional theory (DFT) alters their energetic ordering and produces a new putative global minimum at the DFT level; however, the equilibrium structure predicted by the Gupta potential at room temperature is consistent with previous experiments.

## Abstract 2

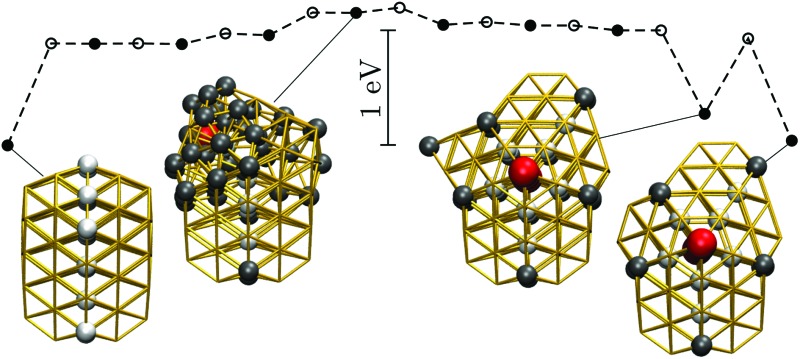
We use the energy landscapes framework to shed new light on the structural diversity of model Au_N_ clusters (30 ≤ *N* ≤ 147), and we find optimal transition pathways connecting prominent morphologies.

## I. Introduction

Gold has been one of the most important elements in cluster science, providing a model system for exploring fundamental questions,^1^,^2^ and offering a range of size- and structure-dependent properties useful in various applications.^3^ Some novel properties, such as the unexpected catalytic activity,^4^ have been linked to specific cluster morphologies, and the perceived structure–property relationship has motivated many studies^5^–^8^ of the atomic-level structure evolution. However, the diversity of morphologies and quasimelting^9^,^10^ observed in gold nanoparticles has hindered systematic understanding, especially in discerning the equilibrium picture and characterising the rearrangement mechanisms at finite temperatures. In the present contribution we use the energy landscapes framework^11^ to shed new light on the polymorphism in model gold clusters.

**Fig. 1:**
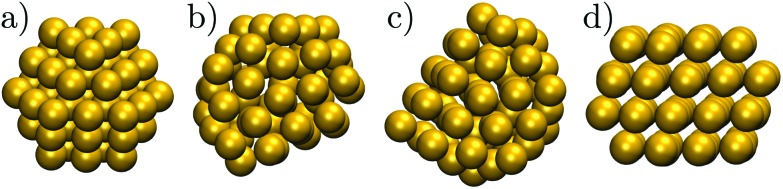
The structure of some Au_55_ isomers from Table 1: (a) Cub; (b) 5; (c) 8; (d) 1.

One of the first and still widely studied gold nanoparticles is the “Schmid Au_55_” cluster,^12^,^13^ formulated as Au_55_ (PPh_3_)_12_ Cl_6_. The Au_55_ core geometry was first characterised as cuboctahedral,^13^,^14^ illustrated in Fig. 1a, with single-crystal face-centred cubic (fcc) atomic ordering. However, this initial interpretation was criticised by Vogel *et al.*,^15^ who found that a model with icosahedral Au_55_ core produced a better fit to the available X-ray powder diffraction data. More recently, Pei *et al.*^16^ used density functional theory to show that many quasi-icosahedral, decahedral, and disordered core structures are energetically favoured over the closed-shell cuboctahedron. To the best of our knowledge, the debate over the Au_55_ core geometry has still not reached a definitive resolution, exacerbating interpretation of the unusual oxidation resistance^17^ and high catalytic activity^18^ of the naked Au_55_ cluster derived from Au_55_ (PPh_3_)_12_ Cl_6_. The intriguing chemistry of the naked Au_55_ may well be at least in part due to a particular geometry,^17^ but the geometry is unlikely to be cuboctahedral, because previous theoretical calculations^19^,^20^ show that the expected lifetime of bare Au_55_ cuboctahedra is too short to be observed under an electron microscope, even in the presence of a substrate.^19^

In another theoretical contribution, Garzón *et al.*^21^,^22^ found several amorphous low-energy structures for the naked Au_55_, with “amorphous” signifying distorted icosahedral order (see Fig. 1b and c), which can exhibit a degree of chirality^23^ and enantioselective properties.^24^ The lowest-lying amorphous isomer (Fig. 1b) was proposed as the global minimum (GM). Indeed, distorted^25^ or amorphized^10^ icosahedra and the partially chiral^8^ Au_55_ structures have been observed under an electron microscope. However, using the same empirical model for Au_55_ as Garzón *et al.*,^21^ Bao *et al.*^26^ found a lower energy structure with fcc order, shown in Fig. 1d, which had previously^27^ been identified for a different interatomic potential. These theoretical predictions suggest that microscopy studies may not be accessing the lowest energy structure, perhaps due to finite temperature effects, an underlying substrate,^28^ the electron beam, or some other factors. Recently,^29^ we performed global optimisation of Au_55_ for a different parametrisation^30^ of the model used by Garzón *et al.*^21^ and Bao *et al.*,^26^ and again found the GM to be fcc, with amorphous isomers dominating at finite temperatures, suggesting that thermal effects are indeed contributing to the discrepancy. In the present study we take a closer look and find that the mean occupation probability of the chiral isomer observed by Wang and Palmer^8^ is actually comparable to the equilibrium occupation probability of the model bound by the same potential as in ref. 21 and 26.

Prediction of an fcc GM for Au_55_ by different empirical models has not yet been adequately tested at higher levels of theory. While DFT has been used to show that the lowest-lying amorphous^22^ and fcc^31^ isomers are individually more stable than other structures, as far as we know the two isomers have not been compared directly using the same DFT functional. Hence, the question of what is the true ground-state morphology of naked Au_55_ still remains unresolved from a theoretical viewpoint. We address this issue through a more comprehensive exploration of structures and direct comparison between different levels of theory, producing a new putative GM for Au_55_ at DFT level. We also verify that high-symmetry morphologies (such as the Mackay icosahedron,^32^ Ino decahedron,^33^ and cuboctahedron) with closed geometric shells are not as stable as one might expect. Depending on the level of theory used, this destabilisation of high-symmetry structures has been linked to either the range of the interatomic potential^21^,^34^,^35^ or relativistic effects.^31^,^35^

Note that identifying the GM is necessary for an accurate description of the equilibrium behaviour within a given model, which is why global optimisation of gold (and other metal) clusters remains an active area of research.^36^ However, the GM alone is not sufficient to explain the finite-temperature behaviour and morphological changes observed in experiments^5^,^7^,^8^ and simulations.^29^,^37^ In fact, finite-system analogues of solid–solid phase transitions have been reported for many metals,^38^,^39^ sometimes well below the size-dependent^1^ melting temperature, but systematic theoretical analysis of this phenomenon for a range of cluster sizes has been performed only for Lennard-Jonesium.^40^,^41^ This omission is partly due to technical difficulties, because the relatively long time scales associated with morphological rearrangements below the melting temperature range cannot be easily accessed using conventional simulation methods. The energy landscapes^11^ framework, on the other hand, provides a powerful approach to studying such rare events, complementing more conventional methods. This framework combines a variety of optimisation and search techniques, statistical mechanics, unimolecular rate theory, and often exploits the harmonic approximation to describe the global thermodynamics and kinetics of complex systems such as atomic clusters.

In the present contribution we explore the potential energy landscape of model Au_N_ clusters (30 ≤ *N* ≤ 147), focusing on their equilibrium thermodyamics in the harmonic superposition approximation,^42^ which accounts for configurational and vibrational entropy. We identify a number of solid–solid transitions in morphology, which arise from the competition between close-packed (single-crystal fcc or lamellar twinned), decahedral, and distorted icosahedral motifs. In selected representative cases we also find the fastest transition pathway between competing motifs. These pathways rarely exhibit the highly cooperative rearrangements identified in other systems,^43^,^44^ but rather involve more localised distortions and are reminiscent of the melt-freeze scenario described by Koga *et al.*^7^

The outline of this paper is as follows: in section II we define the Gupta potential, describe the relevant methods from the energy landscape framework (including molecular dynamics simulation), detail our approach to classifying atomic-level structure, and give the methodological details of our DFT calculations. All the results are discussed in detail in section III, where we first focus on the structure and thermodynamics of the naked Au_55_ cluster, then examine other cluster sizes, and in the end elucidate some rearrangement mechanisms for selected cases. A summary of key findings and conclusions is given in section IV. For completeness, in ESI† we provide: (i) a database of local minima on the Gupta potential energy landscape for all the Au_N_ clusters considered, including the coordinates of the putative global minima; (ii) kinetic transition networks for *N* = 55, 85, and 147; and (iii) the coordinates for the low-lying Au_55_ structures reoptimised using DFT.

## II. Model and methods

We model the binding in Au_N_ clusters of size *N* using the Gupta potential^30^,^45^,^46^
1
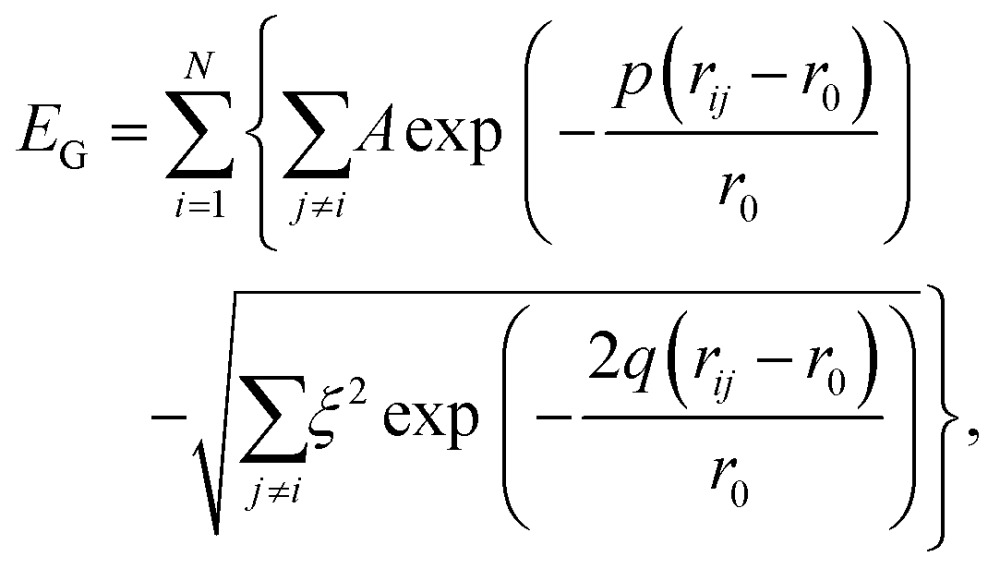
where *r*_*ij*_ is the distance between atoms *i* and *j*, and the remaining parameters are *ξ* = 1.855 eV, *A* = 0.2197 eV, *p* = 10.53, *q* = 4.30, and *r*_0_ = 2.88 Å, as in ref. 35. Note that (1) is based on the second moment tight binding approximation, which remains a standard choice for atomistic modelling of metal nanoparticles,^47^ and in the present study the potential is not truncated (hence no cutoff radius).

### A. Harmonic superposition approximation

To explore the underlying potential energy landscape for a given *N*, we first perform basin-hopping^48^,^49^ global optimisation aided by systematic surface refinement,^50^ with atom-vacancy swap candidates scanned in parallel (as implemented in GMIN^51^). More than 10^4^ lowest-lying minima accumulated during global optimisation provide the input for a harmonic superposition analysis of the thermodynamics.^42^ This analysis involves writing the canonical partition function as2
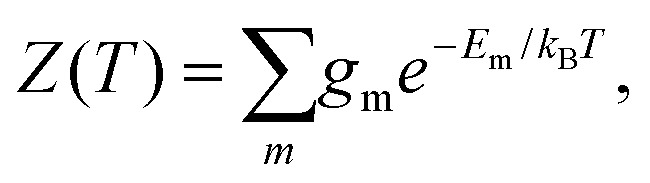
where the sum runs over the local minima, *E*_m_ is the potential energy of minimum *m*, and *k*_B_ is the Boltzmann constant. The degeneracy factor^11^,^29^
3
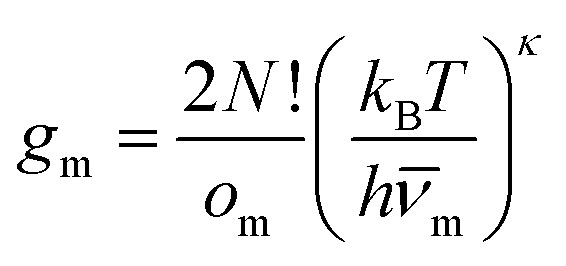
subsumes entropic contributions, represented by the point group order (*o*_m_) and the geometric mean normal mode vibrational frequency (*ν̄*_m_). Here, *κ* = 3*N* – 6 is the number of vibrational degrees of freedom, *h* is the Planck constant, and4
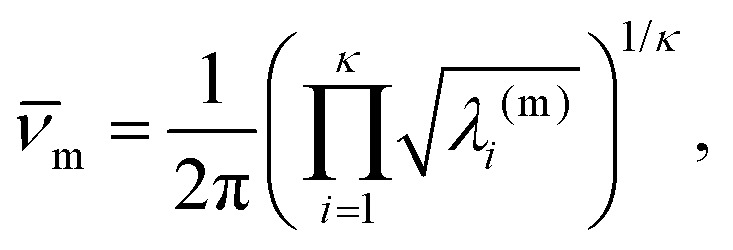
where *λ*_*i*_^(m)^ are the positive, non-zero eigenvalues of the mass-weighted Hessian (the dynamical matrix) for minimum *m*. The occupation probability of each minimum is given by

5 *p*_m_(*T*) = *g*_m_*e*^–*E*_m_/*k*_B_*T*^/*Z*(*T*),

and the vibrational contribution to the heat capacity is6
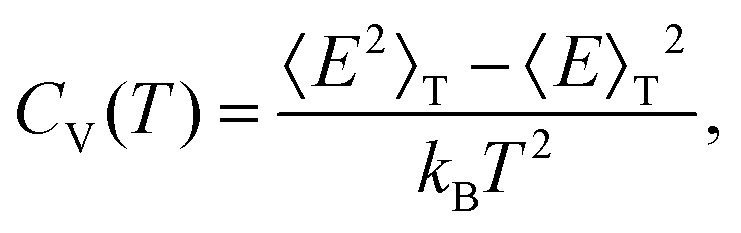
where 
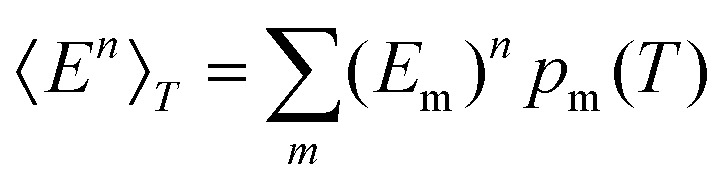
, and the constant kinetic contribution has been omitted. Since the *p*_m_ are additive, we can define collective occupation probabilities 
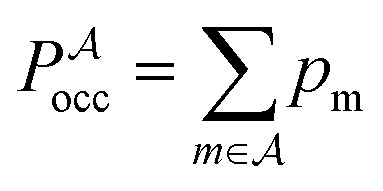
, where 
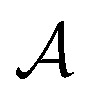
 is a subset of minima.

### B. Kinetic transition networks

For selected cluster sizes we construct a kinetic transition network, whose nodes represent local minima on the potential energy landscape, with edges connecting pairs of adjacent minima separated by a single intervening transition state. Pairs of low-lying minima obtained from global optimisation are first systematically connected using a Dijkstra-based approach^52^ implemented in OPTIM.^53^ The pairwise connection attempts are performed with the doubly-nudged^54^ elastic band algorithm,^55^ with the intervening transition state candidates tightly converged using hybrid eigenvector following,^56^ and intervening local minima converged using the limited-memory Broyden–Fletcher–Goldfarb–Shanno (LBFGS) algorithm of Liu and Nocedal.^57^ The resulting database of stationary points is further extended using discrete path sampling,^58^ exploiting previously described^59^ strategies implemented in PATHSAMPLE.^60^ We visualise these networks in the form of disconnectivity graphs,^61^ which provide a revealing picture of the landscape topography.

To shed light on the rearrangement mechanisms between competing structures, we consider the possible connecting pathways in the corresponding network and identify the one with the largest contribution to the steady-state rate constant.^58^,^59^ This pathway, referred to as the “fastest” for a given temperature, corresponds to the lowest-energy pathway in the limit of zero temperature.

The kinetic lifetime of individual minima is calculated using harmonic transition state theory.^62^ That is, the escape rate from a minimum *m via* an adjacent transition state *s* is7
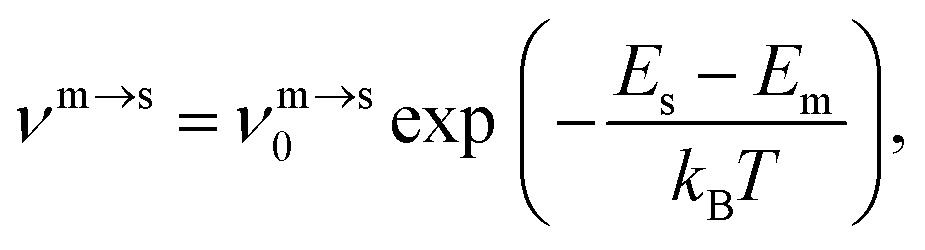
where the pre-exponential frequency factor is given by8
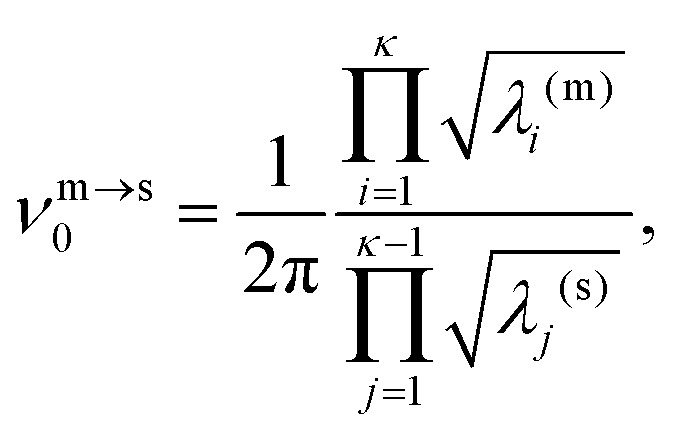
with *i* and *j* spanning the positive (non-zero) eigenvalues of the mass-weighted Hessian matrix evaluated at *m* and *s*, respectively. The temperature-dependent lifetime of *m* is then defined as9
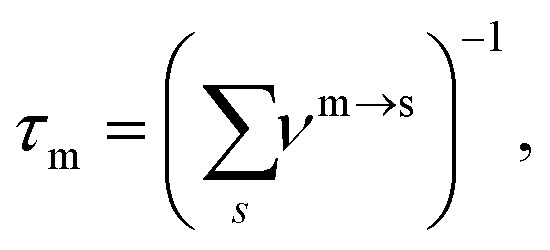
where the sum is over all the directly connected transition states. Note that *ν*_0_^m→s^ can be smaller than *ν̄*_m_ defined in (4), which means that above a certain temperature the lifetime *τ*_m_ will be shorter than the geometric mean period of vibration, signalling a likely breakdown of transition state theory.

### C. Atomic-level structure classification

The atomic-level structure of local minima is characterised using common-neighbour analysis (CNA),^63^ with the nearest neighbours defined by a cut-off distance of 3.5 Å. First, the local order around each atom is classified (using the scheme of Hendy and Doye^64^) as either icosahedral (ico), hexagonal closed-packed (hcp), or face-centred cubic (fcc), with the latter class including well-defined (100) and (111) facets, but not other surface features such as islands, re-entrant grooves, *etc*. Unclassified atoms are simply labelled as ambiguous (amb). We then characterise the overall cluster morphology, *i.e.* the motif, as either icosahedral (ICO), decahedral (DEC), face-centred cubic (FCC), twinned (TWI), hexagonal close-packed (HCP), or otherwise ambiguous (AMB), using a sequence of simple criteria similar to those in ref. 28. First, if an ico atom is neighbour to more than six other ico atoms, then it is regarded as an icosahedral centre and the motif is classified as ICO. Otherwise, if every ico atom is bonded to at most two other ico atoms, and if the total ico atom count is one less than the number of bonds with CNA signature 555, then there is one (local) fivefold symmetry axis (*i.e.* a decahedral spine) and the motif is labelled as DEC. Otherwise, if the number of ico atoms is zero and the combined number of hcp and fcc atoms exceeds the number of amb atoms, then the motif is either FCC if the number of hcp atoms is zero, HCP if the number of fcc atoms is zero, or else it is labelled TWI for “twinned”, though we make no attempt to distinguish between twin planes and stacking faults. Structures failing to satisfy all these criteria are classified as AMB, which is quite a broad category, often capturing minima that are similar to some from the other (more precisely defined) motifs.

### D. Molecular dynamics simulations

To complement our harmonic superposition analysis, the thermal behaviour of selected isomers is simulated using canonical molecular dynamics with a velocity Verlet scheme coupled to a Langevin thermostat.^65^ We used a damping coefficient of 5 ps^–1^ (equivalent to a time constant of 20 fs), and the Verlet integration time step was set to 10 fs. Each simulation ran for 2 × 10^7^ time steps or more, with the first 4 × 10^6^ steps treated as equilibration and discarded. Over the remaining 160 ns we computed the time- and atom-averaged Lindemann index^66^10
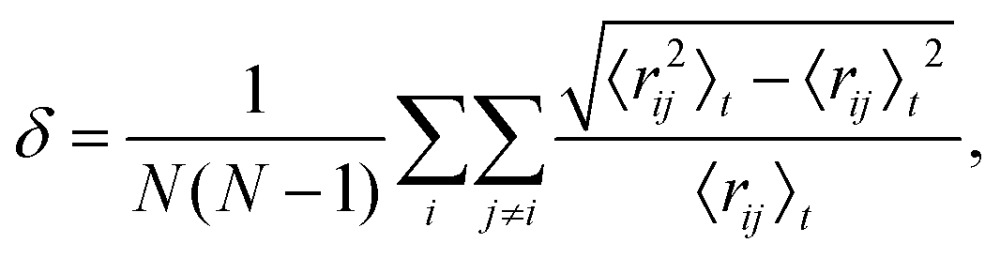
where …_*t*_ indicates a time average of the quantity within the angle brackets. Decreasing the integration time step had no significant effect on *δ*.

### E. Density functional theory calculations

Selected low-lying Au_55_ minima were reoptimised using the Quantum Espresso package^67^—a density functional theory based plane wave code. The exchange–correlation potential was described self-consistently within the generalized gradient approximation (GGA) throughout the Perdew–Burke–Ernzerhof (PBE) functional.^68^ The Rabe–Rappe–Kaxiras–Joannopoulos ultrasoft pseudopotential was used to model the valence electron-nuclei interactions. The energy cut-off for the plane-wave basis set was 40 Ry with a charge density cut-off of 360 Ry. The Au electronic configuration considered was 5d^10^6s^1^. All the calculations were performed at the Gamma point only in a cubic simulation box of at least 29 Å (deemed sufficiently large). Electronic ground state optimisation was performed starting from the ionic structure relaxed at the empirical (Gupta) level. A Gaussian smearing was used with effective electronic temperature of 27.2 meV. The spin–orbit coupling was not included, because it is not expected to have a significant effect on the energy and geometry of Au_55_ minima.^69^

## III. Results and discussion

### A. The naked “Schmid Au_55_”

**Fig. 2:**
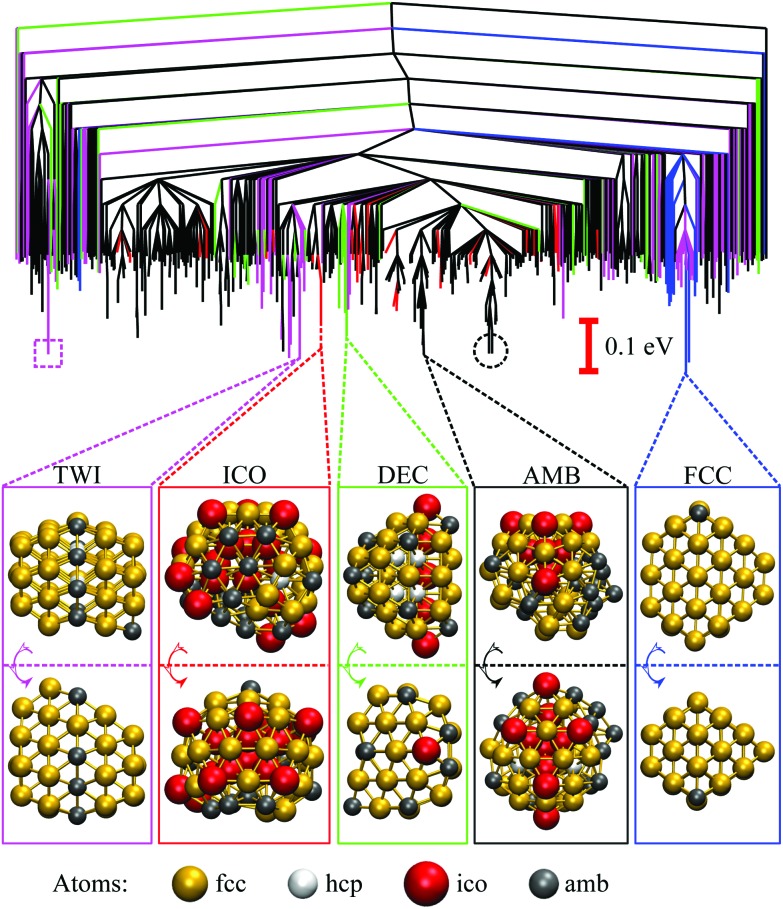
Colour-coded disconnectivity graph for the 500 lowest-lying minima of the Au_55_ cluster. Branches leading to minima of icosahedral (ICO) motif are in red, decahedral (DEC) in green, face-centred cubic (FCC) in blue, twinned face-centred cubic (TWI) in magenta, and the remaining ambiguous (AMB) morphologies in black. The node corresponding to isomer 6 in Table 1 is marked by a square, and isomers 7 and 8 are circled. Ball-and-stick representation of the lowest-lying minimum for each motif is shown from two angles, with atoms colour-coded by the local environment: face-centred cubic (fcc) in gold, hexagonal close-packed (hcp) in white, icosahedral (ico) in red, and ambiguous (amb) in grey. The stick “bonds” are defined by a cut-off distance of 3.5 Å.

The disconnectivity graph for 500 lowest-lying minima of the Au_55_ cluster is shown in Fig. 2. Recall that the vertical axis corresponds to the potential energy, each branch ends at a local minimum, and each node joins minima that can be interconverted without exceeding the energy of the node (thus providing an upper bound on the lowest-energy barrier between them). The energy of the nodes (but not branch endpoints) has been discretised in regular steps for clarity, revealing multiple competing funnels: one narrow funnel comprised of minima with close-packed structure (*i.e.* FCC and TWI motifs), and the others dominated by the AMB motif. Isolated minima of DEC and ICO motifs are also present, and the colour-coded structure of the lowest-lying minimum for each motif is illustrated below the disconnectivity graph. Note that many low-lying ICO and AMB minima resemble icosahedra with discernible triple rosette-like^70^ defects and other surface distortions. However, single and double rosettes are still not particularly favourable, though we expect them to be stabilised by a smaller impurity atom.^29^

More details for the sixteen lowest-lying minima are given in Table 1, confirming the GM found by Bao *et al.*,^26^ and showing that the lowest-lying AMB minimum found by Garzón *et al.*^21^,^22^ is (at best) fifth lowest overall in the given model. The eighth isomer is identified as the chiral structure imaged by Wang and Palmer.^8^ The lowest-lying DEC minimum is fourteenth overall, with the fivefold disclination significantly off centre—in contrast to the Ino^33^ and Marks^71^ decahedra, but reminiscent of some pentagonally twinned structures reported for lead nanoparticles.^72^

**Table 1:** Potential energy relative to the GM (*i.e.* Δ*E* = *E* – *E*^GM^), motif, point group (PG), the geometric mean normal mode vibrational frequencies (*v*) in terahertz, and the number of distinct adjacent transition states (*N*_t.s._) for the sixteen lowest-lying minima and three high-symmetry structures of Gupta Au_55_. Each minimum's occupation probability (*p*_m_) and lifetime (*τ*_m_) in seconds is calculated at room temperature (*k*_B_*T* = 26 meV) using a database of more than 3 × 10^5^ minima and 4 × 10^5^ transition states. The relative energy (Δ*E*_DFT_) of each isomer reoptimised at the DFT level is also given, and the chiral isomer (ranked eighth) imaged by Wang and Palmer^8^ is marked by a dagger

	Δ*E*_G_ (eV)	Motif	PG	*v* (THz)	*N*_t.s._	*p*_m_	*τ*_m_ (s)	Δ*E*_DFT_ (eV)
1	0	FCC	*C*_1_	2.16602	4295	0.0026	2 × 10^–12^	0
2	0.021294	FCC	*C*_s_	2.16593	435	0.0006	3 × 10^–11^	–0.234
3	0.027287	TWI	*C*_1_	2.16607	202	0.0009	8 × 10^–12^	–0.361
4	0.030151	TWI	*C*_1_	2.16641	160	0.0008	1 × 10^–11^	–0.439
5	0.035735	AMB	*C*_1_	2.09791	664	0.1050	2 × 10^–12^	–0.503
6	0.037537	TWI	*C*_3v_	2.16414	230	0.0001	9 × 10^–11^	–0.039
7	0.039020	AMB	*C*_1_	2.09591	276	0.1086	1 × 10^–11^	–0.391
8^†^	0.039037	AMB	*C*_1_	2.09336	555	0.1313	1 × 10^–12^	–0.551
9	0.043134	AMB	*C*_s_	2.13989	259	0.0017	5 × 10^–11^	–0.759
10	0.055655	AMB	*C*_1_	2.09451	176	0.0632	1 × 10^–12^	–0.544
11	0.056596	TWI	*C*_s_	2.16562	198	0.0002	3 × 10^–11^	–0.729
12	0.066701	TWI	*C*_s_	2.16574	86	0.0001	2 × 10^–12^	–0.759
13	0.069885	AMB	*C*_1_	2.12300	125	0.0044	8 × 10^–14^	0.210
14	0.073645	DEC	*C*_s_	2.15466	339	0.0002	2 × 10^–11^	0.449
15	0.088317	AMB	*C*_1_	2.10861	164	0.0062	9 × 10^–14^	–0.500
16	0.093786	ICO	*C*_1_	2.07067	625	0.0899	2 × 10^–11^	0.030

Mac	0.642895	ICO	*I*_h_	1.98361	46	0.0000	1 × 10^–10^	0.644
Ino	1.070310	DEC	*D*_5h_	2.10213	31	0.0000	4 × 10^–14^	1.084
Cub	1.194899	FCC	*O*_h_	2.10972	29	0.0000	5 × 10^–19^	1.748

While the GM structure of Au_55_ is fcc with point group *C*_1_, the symmetric cuboctahedron is significantly higher in energy (by 1.2 eV). This disparity can be explained by differences in surface packing: the 42 surface atoms in the cuboctahedron form only (100) facets, which are known^35^ to be particularly unfavourable in the present model for gold; but in the GM structure the 45 surface atoms are more close-packed and exhibit mainly (111) character. Also, in agreement with previous^21^,^37^ reports of distorted icosahedral order in Au_55_, snapshots of the AMB minima in Fig. 2 exhibit triangular close-packed facets and multiple fivefold disclinations, which outline the tetrahedral units expected in Mackay^32^ icosahedra. The ideal Mackay icosahedron, on the other hand, is not even in the lowest-lying 10^5^ minima (in a database of more around 3 × 10^5^ minima), though it is the most favourable among the closed-shell high-symmetry shapes.

**Fig. 3:**
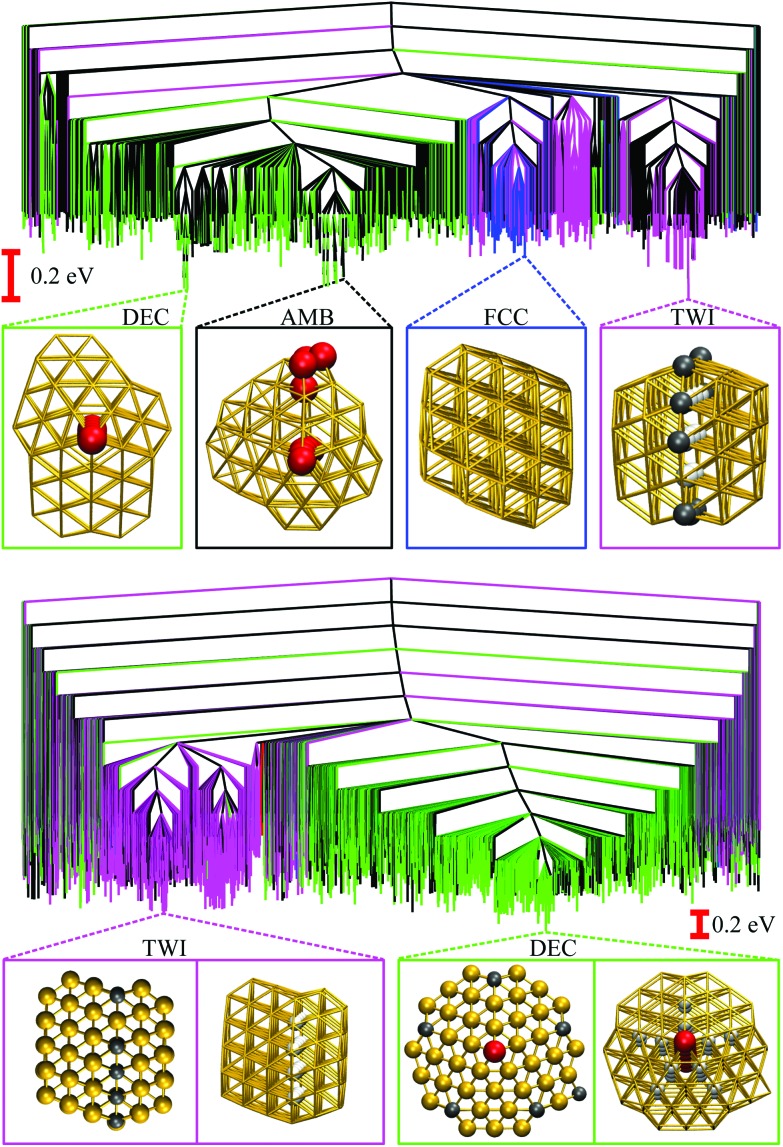
Disconnectivity graphs for 10^3^ minima of Au_85_ (top) and 10^4^ minima of Au_147_ (bottom) with ball-and-stick representations of the lowest-lying minimum for each competing motif. The colour-coding and nomenclature are same as in Fig. 2, and only a subset of atoms is highlighted for clarity.

**Fig. 4:**
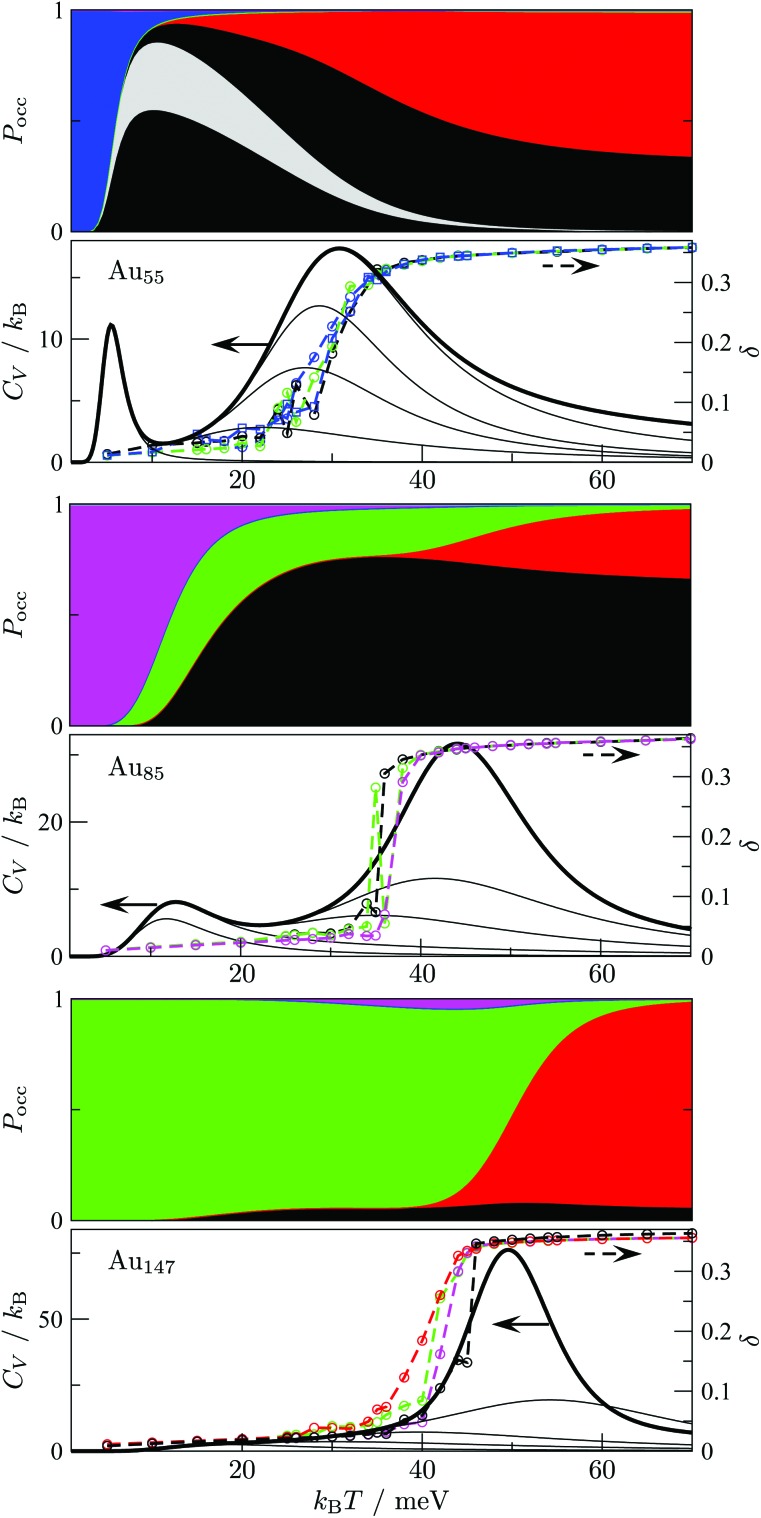
Motif occupation probabilities *P*_occ_ (stacked on top of each other), vibrational heat capacity *C*_V_, and time-averaged Lindemann index *δ* plotted against temperature for Au_55_ (top), Au_85_ (middle), and Au_147_ (bottom). Five motif shares (FCC, AMB, ICO, TWI and DEC) of *P*_occ_ are colour-coded as in Fig. 2 and 3. The chiral (eighth in Table 1) Au_55_ isomer's occupancy is highlighted in grey, splitting the black segment in two, since contributions from individual minima of a given motif are stacked in the order of increasing potential energy. Net *C*_V_ is represented by a thick black line, while thinner black lines correspond to subsets of 10, 10^2^, 10^3^, 10^4^, and 10^5^ lowest-lying minima in our database (sorted by potential energy). The Lindemann index *δ* was calculated from molecular dynamics simulations at temperatures marked by the datapoints, and datasets with the same starting configuration are colour-coded by the initial motif and traced by a dashed line to guide the eye.

The disconnectivity graphs in Fig. 2 and 3 (discussed below) help us to visualise the landscape topography and to identify the funnels associated with competing motifs, but these graphs do not really show how the competition between different morphologies is manifested under thermal conditions. To examine finite-temperature effects we consider the heat capacity *C*_V_ and the collective occupation probability *P*XXXocc of each motif (XXX) as a function of *k*_B_*T* > 0, plotted in Fig. 4.

For Au_55_, *P*FCCocc is the highest below 70 K (*k*_B_*T* < 6 meV). This high occupancy is associated mainly with the GM structure, while other low-lying FCC and TWI isomers combined have near-zero occupation probability at all temperatures, largely due to their low vibrational entropy. *P*AMBocc rapidly grows for *k*_B_*T* in the range 6 ± 1 meV (*T* ≈ 70 K), with several AMB minima reaching comparably high occupation probability, which is illustrated by singling out the contribution from the experimentally observed^8^ chiral isomer (eighth in Table 1 and visualised in Fig. 1c). Recall that a similar transition at a slightly higher temperature (about 90 K) has been identified for a different set of model parameters.^29^ In both cases, when *P*AMBocc supplants *P*FCCocc as the highest value, the crossover temperature coincides with a well-defined peak in the heat capacity. Hence, we characterise the finite-system analogue of a solid–solid like phase transition, where the low-energy phase is represented by a single local minimum of FCC motif, and the high-energy phase comprises multiple AMB isomers. Also note that the absence of magenta and green for Au_55_ in Fig. 4 is due to very low occupancy of DEC and TWI motifs for the entire temperature range considered.

Interestingly, the room-temperature occupation probability of the chiral AMB isomer is 0.13, which is comparable to the occurrence frequency of about 0.1 inferred from experimental data of Wang and Palmer.^8^ This agreement between theory and experiment can be rationalised by taking into account the lifetime of individual isomers (see Table 1). The estimated lifetimes are below a typical vibrational period at room temperature, indicating interconversion among multiple isomers on a timescale significantly shorter than the experimental imaging time. Indeed, Wang and Palmer^8^ acknowledge that their images could be a superimposition of multiple isomers, which would explain why the average occurrence frequency of the chiral isomer observed under the microscope is comparable to the equilibrium occupation probability in Table 1. This rapid fluctuation between several different isomers could also be interpreted as quasi-melting.^9^

Fig. 4 shows that the ICO motif hardly features in Au_55_ at low temperatures, but its occupation probability steadily increases over a temperature range that roughly coincides with a second and more dominant peak in the heat capacity. The time- and atom-averaged Lindemann index *δ* also increases from below 0.1 to above 0.3 in that temperature range, indicating a finite-system analogue of a solid–liquid phase transition (*i.e.* melting). The agreement between MD results and the harmonic superposition approximation is encouraging, with the latter formulation revealing interesting changes in the atomic-level structure of the molten Au_55_. The rise of *P*ICOocc with *k*_B_*T* in the range 30 meV < *k*_B_*T* < 80 meV, with *P*AMBocc decreasing yet remaining significant, suggests a gradually growing preference for local (poly)icosahedral order (*i.e.* increasing fivefold-disclination density) in the melted region.

**Fig. 5:**
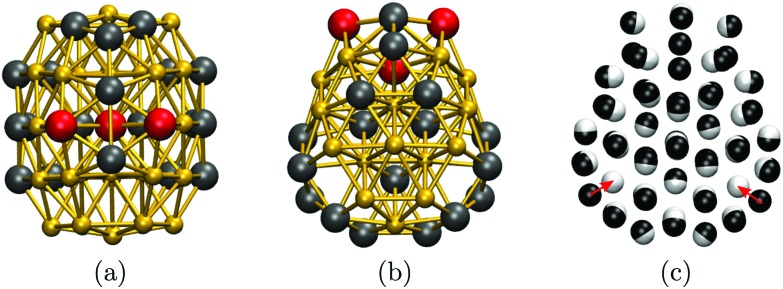
Au_55_ GM structure at DFT level from two (a, b) different viewpoints along the (vertically oriented) symmetry plain, with the ball-and-stick representation colour-coded as in Fig. 2. In (c), the DFT structure (black atoms) is superimposed over the ninth isomer (white atoms) from Table 1, with red arrows highlighting the main difference between the two geometries.

To cross-check our analysis of the Au_55_ cluster, we reoptimised the structure of Gupta minima in Table 1 using DFT. The relaxation produces fairly minor geometric changes, mainly *via* uniform expansion or compression of the isomers. However, there is significant re-ordering of the energies: the fcc GM predicted by Gupta is not even in the ten lowest at the DFT level, where the new putative GM of point group *C*_s_ is obtained by relaxing isomers nine and twelve (in Table 1). The DFT GM structure is shown in Fig. 5, illustrating its amorphous nature and revealing two voids in the subsurface region (see Fig. 5b), consistent with the known propensity of gold clusters to form cage-like structures.^73^ These voids are filled by a nearby surface atom when the geometry is reoptimised for the Gupta potential, as indicated by the two red arrows in Fig. 5c, thus recovering the ninth isomer from Table 1 (with additional and less significant “breathing” of other atoms). Note that, although the two levels of theory do not exactly agree on the GM structure, they both predict the three high-symmetry structures to be very unfavourable, particularly the cuboctahedron. It is also noteworthy that the DFT energy differences (Δ*E*_DFT_ values in Table 1) are an order of magnitude larger than the Gupta energy differences (Δ*E*_G_ values), suggesting that the frustrated nature of the Gupta energy landscape may not be preserved at higher levels of theory.

To conclude our discussion of the naked “Schmid Au_55_”, we calculate the HOMO–LUMO energy gap and the partial density of states for isomers 1, 6, 8, 9, 14, Mac, Ino, and Cub in Table 1. The HOMO–LUMO gap (calculated using the ΔSCF method,^74^ comparing the ground state of charge +1 and –1 with the neutral system, including the Makov–Payne correction^75^) ranges from 2.18 to 2.26 eV among these isomers. The total density of states for the three symmetric isomers (particularly Mac) shows pronounced peaks around specific values, while for the more ambiguous structures the density is more broadly distributed. Unfortunately, partial density of states does not immediately reveal any links between the electronic structure and the cluster geometry, but we hope to explore this issue in more detail as a separate study.

### B. Size dependence

We now consider other Gupta Au_N_ clusters in the size range 30 ≤ *N* ≤ 147, starting with a direct comparison between Au_55_, Au_85_, and Au_147_. The corresponding disconnectivity graphs are shown in Fig. 2 and 3, with the heat capacities and motif-decomposed occupation probabilities plotted in Fig. 4.

As for Au_55_, the ideal Au_147_ Mackay icosahedron^32^ is energetically unfavourable, with the disconnectivity graph based on 10^4^ lowest-lying minima featuring hardly any traces of the ICO motif. The fraction of AMB minima is also reduced (compared to Au_55_) and the existence of two structurally homogeneous funnels is apparent: one is predominantly DEC, and the other is TWI. Note that the GM of Au_147_ is a 146-atom Marks decahedron,^72^ with six atoms along the fivefold disclination (*i.e.* the decahedral spine), and an extra adatom on one of the peripheral (100) facets. The DEC motif is the most populated for *k*_B_*T* ≤ 40 meV (Fig. 4) and is gradually supplanted by the ICO motif in the range 50 ± 10 meV, over which *P*AMBocc and *P*TWIocc also rise up to 0.09 and 0.04, respectively. The onset of the ICO motif is more abrupt than for Au_55_, still coinciding with a *C*_V_ peak, but the corresponding temperature range is noticeably above the range over which *δ* reaches the value of 0.3. This apparent mismatch between molecular dynamics and the harmonic superposition approximation may be due to our database of minima under-representing the melted region, but it could also be due to an harmonic effects.

Au_85_ is in some ways intermediate between Au_55_ and Au_147_. Its disconnectivity graph (see Fig. 3) shows fairly pronounced FCC, TWI, and DEC funnels, and each funnel exhibits a considerable number of AMB minima. In this particular case the distinction between DEC and AMB minima is marginal, because many AMB structures still exhibit a well-defined decahedral spine, albeit with a higher degree of amorphisation and/or locally (poly)icosahedral order at the surface. As a consequence of our motif definitions, the low-temperature *C*_V_ peak for Au_85_ in Fig. 4 straddles two crossover temperatures. At *k*_B_*T* = 13 meV the TWI motif is supplanted by the DEC motif as the most populated, and at *k*_B_*T* = 17 meV it is the AMB motif that starts to dominate. The Lindemann index rises at a noticeably lower temperature than the second *C*_V_ peak, similar to Au_147_, showing that the discrepancy is not specific to a particular cluster size. Interestingly, the increase in *δ* for Au_85_ and Au_147_ seems to better align with the onset of ICO isomers, when *P*ICOocc ceases to be negligible but does not yet dominate, suggesting that the onset of multiple fivefold disclinations and icosahedral cores can be take as an indicator of melting.

From Fig. 4 it is apparent that the maximal value of *P*AMBocc diminishes with cluster size, which is consistent with Bao *et al.*^26^ finding amorphous GM only for *N* < 55. However, our thermodynamic analysis shows that the AMB motif can also dominate in larger clusters at finite temperatures. To explore this avenue further we systematically analysed Au_N_ clusters with *N* = 30–147, accumulating a database of about 10^4^–10^5^ low-lying minima for each *N*. We also determined two crossover temperatures, *T*_A_ and *T*_I_, respectively marking when the AMB and ICO motifs become the most populated. The results are summarised in Table 2, together with an estimate of the melting temperature (*T*_m_) range obtained from molecular dynamics simulations using the Lindemann^66^ index defined in eqn (10).

**Table 2:** The GM energy, motif, and point group (PG) for Gupta Au_*N*_ clusters; two crossover temperatures *T*_A_ and *T*_I_, respectively marking when the AMB and ICO motifs become the most probable; and the melting temperature *T*_m_, estimated from MD simulations for selected sizes using the Lindemann criterion. Note that *T*_A_ = 0 K when the GM motif is AMB, and *T*_A_ and *T*_I_ are N/A when the corresponding motif is never the most probable for *k*_B_*T* ≤ 0.1 eV

*N*	*E*_GM_ (eV)	Motif	PG	*T*_A_ (K)	*T*_I_ (K)	*T*_m_ (K)	*N*	*E*_GM_ (eV)	Motif	PG	*T*_A_ (K)	*T*_I_ (K)	*T*_m_ (K)
30	–104.743100	AMB	*C*_3v_	0	460	290 ± 50	60	–213.536346	TWI	*C*_1_	142	446	370 ± 30
31	–108.183974	AMB	*C*_2_	0	402		61	–217.254337	TWI	*C*_3v_	223	585	
32	–111.794339	AMB	*C*_3_	0	306		62	–220.855915	TWI	*C*_s_	209	504	
33^*a*^	–115.404251	ICO	*C*_1_	193	339		63^*b*^	–224.532317	TWI	*C*_s_	223	316	
34	–119.082484	AMB	*C*_3_	0	295		64	–228.254019	DEC	*C*_2v_	299	397	
35	–122.678712	AMB	*C*_s_	0	330	260 ± 50	65	–231.868903	DEC	*C*_2v_	220	457	370 ± 50
36	–126.240885	AMB	*C*_2_	0	100	280 ± 50	66	–235.547173	DEC	*C*_s_	70	476	
37	–129.991492	AMB	*C*_2v_	0	248	280 ± 50	67	–239.158600	DEC	*C*_s_	207	555	
38	–133.584814	AMB	*C*_s_	0	309	300 ± 50	68	–242.838649	DEC	*C*_2v_	153	935	
39	–137.184370	FCC	*C*_4v_	12	137	300 ± 50	69	–246.450465	DEC	*C*_1_	172	982	
40	–140.789863	AMB	*C*_s_	0	35	330 ± 50	70	–250.154102	DEC	*C*_s_	100	N/A	370 ± 30
41	–144.403059	AMB	*C*_s_	0	262		71	–253.959033	DEC	*C*_2v_	339	N/A	
42	–148.023359	AMB	*C*_1_	0	367		72	–257.571776	DEC	*C*_s_	239	N/A	
43	–151.721379	DEC	*C*_2v_	9	339	320 ± 50	73	–261.253928	DEC	*C*_s_	95	N/A	
44	–155.322847	AMB	*C*_s_	0	260		74	–264.922500	DEC	*C*_5v_	297	N/A	
45	–158.916745	DEC	*C*_2v_	9	422	330 ± 20	75	–268.761948	DEC	*D*_5h_	397	N/A	380 ± 30
46	–162.598451	AMB	*C*_3_	0	487		76	–272.372659	DEC	*C*_2v_	337	N/A	
47	–166.245363	DEC	*C*_2v_	128	441	340 ± 50	77	–275.982269	DEC	*C*_2v_	123	N/A	
48	–169.873475	AMB	*C*_1_	0	471		78	–279.588677	DEC	*C*_2v_	158	N/A	
49	–173.562095	DEC	*D*_5h_	111	513		79	–283.417486	FCC	*O*_h_	385	N/A	420 ± 30
50	–177.090944	TWI	*D*_3h_	35	520	330 ± 20	80	–287.022207	FCC	*C*_4v_	265	N/A	410 ± 30
51	–180.698557	AMB	*C*_1_	0	457		81	–290.660813	DEC	*C*_2v_	169	N/A	
52	–184.431342	AMB	*C*_2v_	0	545		82	–294.269106	DEC	*C*_2v_	232	N/A	
53	–188.009110	AMB	*C*_3v_	0	552		83	–297.933795	TWI	*C*_s_	276	N/A	
54	–191.686569	FCC	*C*_2v_	14	404		84	–301.598992	DEC	*C*_s_	181	N/A	
55	–195.284851	FCC	*C*_1_	70	501	350 ± 20	85	–305.259030	TWI	*C*_2v_	197	N/A	420 ± 30
56	–199.015100	FCC	*D*_2h_	165	344		86	–308.936312	DEC	*C*_2v_	318	N/A	
57^*c*^	–202.624795	TWI	*C*_2v_	176	534	360 ± 50	87	–312.550788	DEC	*C*_2v_	367	N/A	
58	–206.216538	TWI	*C*_2_	42	483		88	–316.280622	FCC	*C*_s_	327	N/A	
59	–209.852603	TWI	*C*_2v_	153	295		89	–319.884977	FCC	*C*_s_	443	N/A	

120	–434.088354	DEC	*C*_s_	624	N/A	470 ± 20	90	–323.613329	FCC	*C*_s_	436	N/A	420 ± 20
140	–508.052209	DEC	*C*_s_	778	N/A	500 ± 30	95	–341.874115	TWI	*C*_s_	309	N/A	420 ± 30
147	–533.942249	DEC	*C*_s_	N/A	590	490 ± 30	100	–360.342412	FCC	*C*_1_	343	N/A	450 ± 30

^*a*^Low-lying isomers of borderline ICO/AMB motif produce reentrant behaviour and a particularly broad heat capacity peak.

^*b*^The GM motif is first supplanted by the DEC motif, and then by the AMB motif at temperature ***T***_**A**_.

^*c*^The lowest-lying ICO isomer is borderline and has been reclassified as AMB to eliminate artificial reentrant behaviour.

Table 2 shows that the GM structures are predominantly AMB for *N* < 54, then primarily FCC or TWI in the size range 54 ≤ *N* < 64, and mostly DEC in the range 64 ≤ *N* < 88, which includes the closed-shell Marks decahedron^71^ for *N* = 75. Note that AMB and DEC motifs are in close competition for *N* between 42 and 49, as discussed in more detail in section IIIC, while the size range 88 ≤ *N* ≤ 147 shows re-entrance of the FCC/TWI and DEC motifs. As cluster size increases, the GM motif changes in the sequence AMB → FCC/TWI → DEC → FCC/TWI → DEC → …, which differs from the generally expected^41^,^76^ sequence ICO → DEC → FCC/TWI. Furthermore, although the AMB motif is the GM only for *N* < 54, we find that *T*_A_ < *T*_m_ and *T*_A_ < *T*_I_ for most of the cluster sizes considered, and so amorphous structures still dominate for *N* ≥ 54 at temperatures between *T*_A_ and min{*T*_m_, *T*_I_}. Also note that *T*_I_ > *T*_m_ or *T*_I_ is N/A in many cases, which implies general thermodynamic instability of well-defined icosahedral order in the solid state.

In passing we confirm that the GM of Au_38_ is a low-symmetry AMB isomer, as discovered by Garzón *et al.*,^22^ with the symmetric truncated octahedron (of FCC motif) higher in energy by 6 meV. The ordering is reversed when one extra atom is added: the GM of Au_39_ is FCC, comprising the 38-atom truncated octahedron with the extra atom placed on one of the (100) facets, essentially converting the facet into a vertex. This behaviour is again consistent with energetically unfavourable (100) facets,^35^ making Au_39_ the smallest cluster with a single-crystal fcc GM, which beats the next-lowest AMB isomer by less than 2 meV. Given these small energy differences it is reasonable to expect the ordering to vary for different models and levels of theory.

### C. Geometric odd–even behaviour

**Fig. 6:**
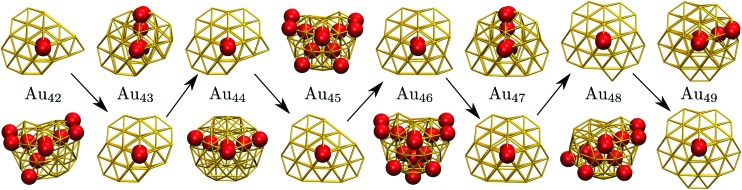
The GM (bottom row) and the lowest-lying minimum of a different motif (top row) illustrated for Au_*N*_ clusters in the size range exhibiting odd–even behaviour, with the GM alternating between AMB and DEC motifs. Red spheres represent ico atoms and the arrows keep track of the DEC motif with four atoms along the decahedral spine.

Interestingly, near degeneracy of the AMB and DEC motifs in the size-range 42 ≤ *N* ≤ 49 causes the GM structure to alternate, with the DEC motif prevailing for odd *N*. This behaviour is illustrated in Fig. 6, providing an interesting example of geometric odd–even effect in atomic clusters. The lowest-lying DEC isomer is *not* the GM for even *N*, when there is an unpaired adatom on one of the peripheral (100) facets of the incomplete Marks decahedron. Although this odd–even effect is unlikely to be general, and indeed it is not as pronounced in Sutton–Chen clusters^77^ (breaking down for *N* = 45), it nonetheless illustrates a more extreme case of the reentrant phase behaviour reported for silver clusters.^78^ We also note that in several cases (*e.g. N* = 43, 47, and 49) the structural differences between the lowest-lying DEC and AMB minima seem fairly minor: the AMB isomer still exhibits a discernible decahedral spine, which is slightly crooked due to more localised fivefold disclinations near the surface.

**Fig. 7:**
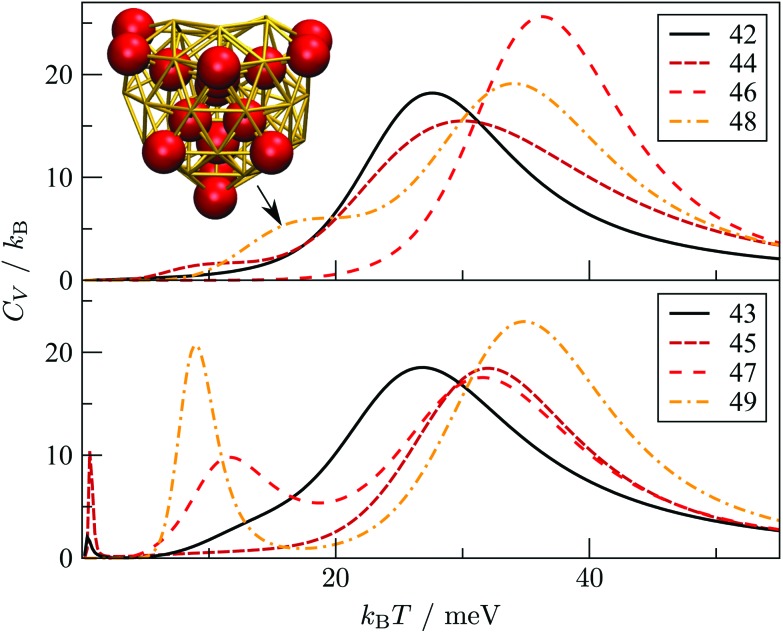
Heat capacity (*C*_V_) *versus* temperature (*k*_B_*T*) for Au_*N*_ clusters with even (top) and odd (bottom) *N* in the range 42 ≤ *N* ≤ 49. For Au_48_, the indicated *C*_V_ shoulder corresponds to the isomer illustrated in the inset supplanting the GM shown in Fig. 6.

To check that the range of odd–even behaviour in Table 2 is not an artefact of our motif definitions, but actually has thermodynamic implications, we consider the heat capacity in the size range 42 ≤ *N* ≤ 49. Fig. 7 shows two well-defined *C*_V_ peaks featuring for odd *N*, one near the temperatures *T*_A_ and the other closer to *T*_I_. The low-temperature peak in each case marks the crossover from DEC to AMB motif, while the high temperature peak straddles the melting range and a crossover from the AMB to the ICO motif as the most populated. The low-temperature peaks are absent for even *N*, consistent with *T*_A_ = 0. The results for odd *N* = 43, 47, and 49, on the other hand, show that thermally activated manifestation of seemingly minor local icosahedral order near the surface of a cluster can produce a well-defined peak in the heat capacity.

### D. Vibrational entropy and fivefold disclinations

Although the *C*_V_ plots in Fig. 7 do not exhibit a pronounced low-temperature peak for even *N*, there are still some identifiable premelting features in the form of a low-temperature shoulder to the main (melting) peak. These features arise from inhomogeneities within the AMB motif. For instance, the low-temperature shoulder for Au_48_ marks a transition from the GM (see Fig. 6) to a higher energy minimum (see Fig. 7 inset) of the same AMB motif, but with considerably more ico atoms. The highest ico coordination of ico atoms is below seven in both minima, which is why they are both classified as AMB. Hence, there is scope for splitting the AMB motif into finer submotifs, defined by additional criteria on the number and topology of ico atoms, or using a more general approach^79^ based on the temperature derivative of occupation probabilities for individual minima, which can help better interpret various *C*_V_ features. In the present model we generally observe that vibrational entropy tends to be larger for minima with more ico atoms, indicative of normal modes softening with increasing fivefold disclination density. This observation explains why thermally activated morphological transitions generally follow the order FCC/TWI → DEC → AMB → ICO, which corresponds to increasing number of ico atoms and increasing vibrational entropy. Note that equilibrium transitions of type FCC/TWI → DEC occur for *N* = 63, 79, 83, 85, 89 and 90, as indicated by the superscript b in Table 2.

### E. Rearrangement mechanisms

**Fig. 8:**
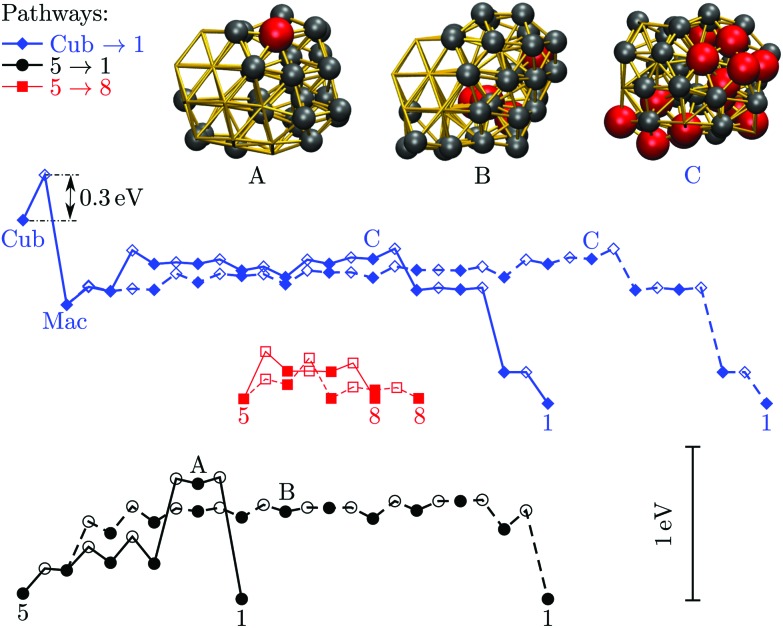
Potential energy profiles of the fastest discrete pathway between selected endpoints (isomers Cub, 1, 5 and 8 from Table 1). The horizontal and the vertical axes correspond to the (Gupta) potential energy and the discrete path length, respectively, with the same scaling for all the profiles. Solid lines trace pathways for *k*_B_*T* = 26 meV (room temperature) and dashed for 5 meV. Filled and unfilled symbols correspond to minima and saddles, respectively. The structure of three minima (labelled A, B and C) is shown with the amb and ico atoms highlighted and colour-coded as in Fig. 2.

We now consider the rearrangement mechanisms for selected morphological transitions, focusing on the fastest transition pathways in Au_55_, Au_85_ and Au_147_. We start with the pathway connecting the symmetric cuboctahedral isomer of Au_55_ with the fcc GM. Given that both endpoints are single-crystal fcc, our intuition suggests a transition pathway based on a sequence of atom-hops on the cluster surface. However, it turns out that the fastest route in and out of the cuboctahedral local minimum is *via* a particular transition state of point group *T*_d_, corresponding to the diamond–square–diamond mechanism,^43^ which connects the cuboctahedron to the closed-shell Mackay icosahedron.^20^ The complete pathway is illustrated in Fig. 8, showing how the intervening Mackay icosahedron rearranges into the fcc GM *via* a sequence of local minima with no discernible order.

Although the lowest-lying AMB isomer is only 36 meV higher in energy than the GM, the lowest overall energy barrier is about 0.5 eV, and the corresponding pathway involves minima with partially disordered geometries, such as snapshot B in Fig. 8. Hence, if this transition is observed, it would most likely resemble partial melting followed by crystallisation into the fcc structure. Interestingly, the maximal degree of amorphisation along the fastest path decreases as the temperature increases, which can be seen by comparing the fastest pathways at *k*_B_*T* = 26 meV and 5 meV. This somewhat counter-intuitive trend is not unexpected considering that most low-lying minima of Au_55_ exhibit fairly amorphous or ambiguous structure (recall Fig. 2). It is also noteworthy that the number of steps in the fastest pathway is more than halved when the temperature is increased from *k*_B_*T* = 5 meV to room temperature (26 meV), showing that the fastest mechanism is temperature dependent.

Fig. 8 also shows the potential energy profile for the optimal pathway between isomers five and eight in Table 1 at two different temperatures. The profiles involve considerably lower energy barriers compared to the other pathways, consistent with the disconnectivity graph in Fig. 2, and showing no significant change with temperature.

**Fig. 9:**
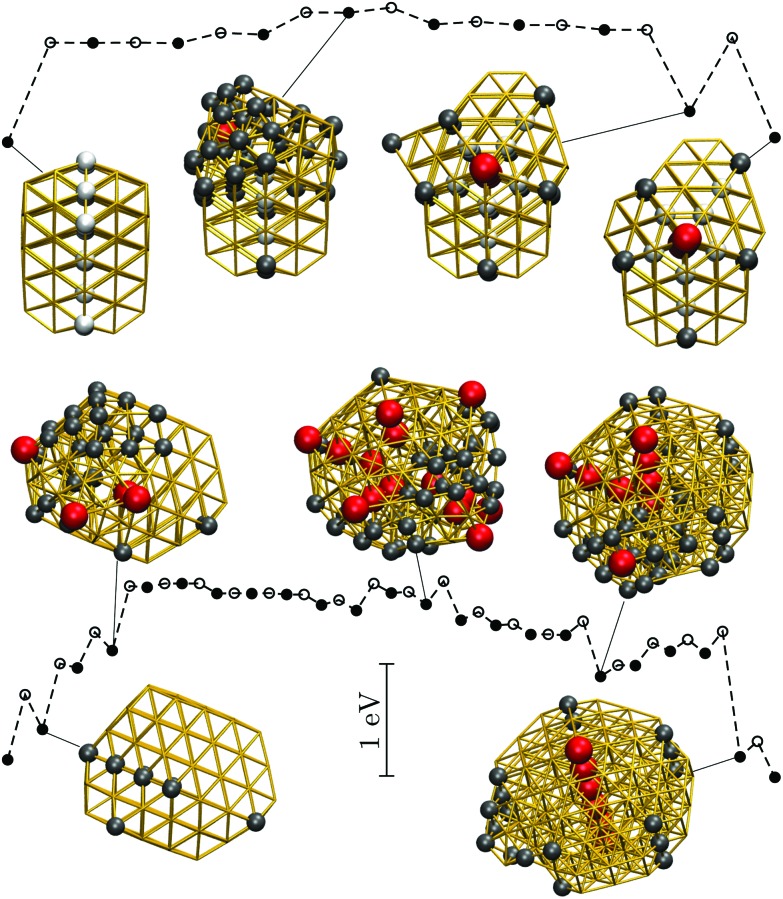
Potential energy profiles for the fastest discrete pathway from the lowest-lying TWI minimum (leftmost) to the lowest-lying DEC minimum (rightmost) for Au_85_ (top) and Au_147_ (bottom) at room temperature (*k*_B_*T* = 26 meV). The horizontal and the vertical axes, the meaning of filled/unfilled symbols, and the colour-coding of the atoms in the selected snapshots are as in Fig. 8.

Recall that the most competitive motifs for *N* > 56 at low temperatures are DEC and TWI, so we examine if the fastest pathway between these two motifs follows any particular mechanism, using Au_85_ and Au_147_ at room temperature as our test cases. For Au_85_, Fig. 9 shows that part of the twin boundary in the lowest-lying TWI isomer is preserved along the pathway to the lowest-lying DEC isomer. However, formation of the fivefold twin axis is accompanied by a significant level of disorder, which would probably be interpreted as partial melting if observed in experiments. A more significant level of disorder occurs in the Au_147_ pathway (see Fig. 9), where (unlike Au_85_) the amorphous intermediates exhibit multiple well-defined fivefold disclinations, somewhat resembling a distorted icosahedron. Most of these amorphous intermediates are about 1 eV higher in energy than the TWI and DEC endpoints, while among themselves they are separated by relatively small energy barriers, which also suggests that the calculated pathway passes through a liquid-like state.

## IV. Summary and conclusions

We have applied the energy landscapes framework to map the equilibrium morphologies of Au_N_ clusters in the size range 30 ≤ *N* ≤ 147, modelled using the Gupta potential, and we examined the rearrangement mechanisms between some competing structures. Our results complement previous theoretical and experimental studies and shed new light on finite-size and temperature effects.

The global minimum (GM) structure of most clusters in the size range 30 ≤ *N* ≤ 53 was found to be fairly ambiguous, but with discernible fivefold disclinations. We identified a case of geometric odd–even behaviour in the range 42 ≤ *N* ≤ 49, where the GM alternates between a structure with just one disclination and a structure with multiple disclinations. Global minima of larger clusters (54 ≤ *N* ≤ 147) are typically a lamellar-twinned or single-crystal fcc lump, or a decahdral motif with a single fivefold disclination. While this structural change is consistent with previous global optimisation studies,^26^ our thermodynamic analysis further shows that ambiguous motifs with multiple fivefold disclinations can still dominate for *N* ≤ 85 (except *N* = 71, 75, 76, and 79) at around room temperature.

In most cases where the GM has at most one disclination, we found thermally-driven morphological transitions below the size-dependent melting temperature. These finite-system analogues of a solid–solid phase transition often coincide with a premelting feature in the heat capacity curve, and they typically correspond to the GM occupation probability dropping below that of multiple ambiguous structures above a system-specific temperature. In certain cases (*N* = 63, 79, 83, 85, 89, 90) we found two consecutive premelting transitions between distinctly different motifs, with the corresponding crossover temperatures correlating with a smeared shoulder or peak in the heat capacity. In all cases, the higher-energy phase exhibits more fivefold disclinations and higher vibrational entropy.

We calculated the fastest solid–solid transition pathways for Au_N_ with *N* = 55, 85, 147, where the energy barrier separating some of the competing motifs is up to 1 eV. While such energy barriers are difficult to treat using conventional simulation methods, which makes direct simulation of the rearrangement mechanism unfeasible, discrete path sampling and harmonic transition state theory provide a useful approximation that performs best at low temperatures. The calculated pathways pass through many metastable intermediates with fivefold disclinations and/or a high degree of amorphisation, consistent with the melt-freeze scenario described by Koga *et al.*^7^ for larger gold clusters.

Finally, we confirmed a previously reported^26^,^29^,^80^ fcc GM structure of point group *C*_1_ for Au_55_, but our thermodynamic analysis revealed that several distorted icosahedra collectively become more favourable at temperatures above 50 Kelvin. Interestingly, the room-temperature occupation probability of a particular isomer was found to be consistent with electron microscopy observations of Wang and Palmer.^8^ This apparent agreement is surprising, because the Gupta potential energy landscape for Au_55_ clearly disagrees with density functional theory (DFT): relaxing the geometry of sixteen lowest-lying Gupta minima using DFT produced a markedly different energetic ordering, with a new putative GM of point group *C*_s_ at the DFT level. While the consistency between our Gupta-level calculations and previous experiments may well be fortuitous, it nonetheless highlights the importance of accounting for thermal fluctuations in geometry—something that is often overlooked when comparing empirical potentials with DFT. In future studies it would be interesting to investigate the equilibrium thermodynamics of Au_55_ (and other clusters) at the DFT level, where it is also possible to compute the normal-mode frequencies for the harmonic superposition approximation.

## Conflicts of interest

There are no conflicts to declare.

## Supplementary Material

Supplementary information
